# Experiences of family members of relatives admitted as state patients in a psychiatric hospital

**DOI:** 10.4102/sajpsychiatry.v29i0.1958

**Published:** 2023-07-26

**Authors:** Seipati Malebye, Nompumelelo Ntshingila, Marie Poggenpoel

**Affiliations:** 1 Department of Nursing, Faculty of Health Sciences, University of Johannesburg, Doornfontein, South Africa

**Keywords:** admitted, experiences, family members, relative, state patient, qualitative

## Abstract

**Background:**

Family members play an important role in caring for state patients during their admission to a psychiatric hospital. They receive limited support from the multidisciplinary team because they do not have a relationship that will promote the families to verbalise their thoughts, rather the interaction that the multidisciplinary team and family members share is about the admitted state patient.

**Aim:**

This article explored and described the experiences of family members who have a relative admitted as a state patient in a psychiatric hospital. Based on the findings, specific recommendations were provided to facilitate the mental health of state patients’ family members in the future.

**Setting:**

The study was conducted in participants’ homes; only one interview took place in the psychiatric hospital when the family member came to meet the multidisciplinary team.

**Methods:**

The study employed a qualitative, exploratory and contextual research design. Family members’ lived experiences were explored using in-depth phenomenological interviews and later analysed.

**Results:**

The findings indicated family members experienced negative feelings, a sincere desire to support their relatives and a great need to share information and knowledge about mental illness.

**Conclusion:**

The study indicated that state patients’ family members’ mental health should be focused on to improve their understanding of mental illness.

**Contribution:**

The findings of this study call for collaboration between the family members, the police and multidisciplinary teams from the hospitals, the mental health awareness and counselling for families.

## Introduction

Family members’ experiences of having a relative admitted as a state patient are not fully understood. They do not interact with the multidisciplinary team to educate them about mental illness and unlawful acts leading to state patients. *South African Criminal Procedure Act*, No. 51 of 1977^1^ section 77 (6)(a)(i) and (ii) do not allow persons who have a mental illness or intellectual disability to be charged with criminal offences because they would not be able to understand criminal proceedings. This also means they will not serve time in prison but might be admitted to a psychiatric institution for care, treatment and rehabilitation. Furthermore, they will be referred to as state patients.^2^ The *South African Criminal Procedure Act*, No. 51 of 1977^1^ governs state patients’ admission for mental healthcare, treatment and rehabilitation in South Africa, under section 42 of the *Mental Health Care Act*, No. 17 of 2002.^2^

Family members play an essential role in caring for state patients during their admission to a psychiatric hospital.^3^ They have a duty to remain in constant contact with their relatives and show support as inadequate support contributes to institutionalisation.^4^ Furthermore, family members should attend to the multidisciplinary team’s (MDT) needs and prepare their mindset and the community for their relative’s possible return.^4^ Research^4^ found that when a state patient is admitted to a psychiatric hospital, their family members experience feelings of relief and guilt that their relative has been taken away; however, the admission does not imply a long-term solution and the relatives will at some point return home. Relatives are often discharged either conditionally, unconditionally or reclassified as involuntary patients.^2,5^ This indicates that family members require support.

To the researcher’s observations, family members (often the primary caregivers) do not have information about mental illness. Wankiiri^3^ added that the lived experiences of families with a mentally ill relative are unknown, yet they play an important role in caring. Family members face severe stigmatisation from the community when their relative has a mental illness and criminal history.^6^ Even when community members learn that mental illness led to the offence, they view these individuals as criminals rather than persons with mental health needs.^7,8^ Studies also indicate that family members caring for a mentally ill relative might suffer from guilt, fear and burden, yet little attention is being paid to them and their mental well-being is not being attended to.^9^ Mental health is defined by how people act, feel and cope or act with their experiences of adversity.^10^ It has been determined that families experience serious emotional burden such as feelings of hopelessness and social burdens looking at stigmatisation from the community because of having a state patient relative, which jeopardises their mental health.^11^

Mothwa^4^ conducted a South African study addressing the challenges families experienced while caring for state patients. It was identified that for families’ needs to be met, the MDT had to understand their views and challenges and the strengths that enabled them to cope. Support groups for family members of relatives admitted as state patients appeared to have a positive effect as families shared their perceptions and strengths with one another.^12,13^ Some families also wanted their relatives to be hospitalised for relief because of the violence they experienced when their relative was mentally ill.^14^ McCabe^15^ determined family members often had good relationships with the MDT when their interaction was about rehabilitating their relative. They also became motivated after seeing their relatives’ progress or positive change.

In South Africa, forensic psychiatry has several issues. These include a scarcity of forensic psychiatric institutions and a scarcity of qualified psychiatrists working in forensic psychiatry in the public sector.^16^ Sociopolitical and socioeconomic variables, such as high crime levels, poverty, inequality, unemployment and many shortcomings of the frequently chastised South African Police Service (SAPS), pose additional obstacles as they encourage non-adherence and weak social support.^17^ In the Free State province, between 2000 and 2004, there were 71 state patients, and most were males.^18^ Another study conducted at Sterkfontein Hospital in Gauteng province indicated that 117 state patients were admitted during the study period between 01 January 2004 and 31 December 2005.^17^

This article aimed to explore and describe the experiences of family members who have a relative admitted as a state patient. Their mental state regarding emotions, social interaction, and thinking were explored. Recommendations of specific measures will be made to improve the mental health of state patients’ family members.

## Theoretical framework

The study is based on the Kubler-Ross Model, which explains the stages of grief.^19^ This model addresses how individuals cope with grief and loss. In this study, the focus is on mental illness. It has been suggested that individuals respond in an almost similar manner when they are faced with serious life-changing events, therefore, these stages neither occur in this sequence nor would an individual experience them all. They experience denial as the first response by not accepting the changes they face. Then follows anger when the life changes persist to the worst with no chance of becoming better. Anger is directed at people around, and there might be blame. Bargaining is the next stage, where an individual would feel to exchange mental illness for something less severe. Depression comes as a fourth stage, where individuals are sad about the realities of living with mental illness or living with the results of mental illness. Some may feel suicidal or hopeless. The last stage is acceptance, when emotions stabilise and learning to live with a new reality of having a state patient within the family.

## Research methods and design

A qualitative, exploratory and contextual research design was employed.^20^ Exploration in qualitative research helps researchers to gain new meaning and knowledge about the phenomenon under investigation. The researcher explored family members’ experiences by asking questions that provided an opportunity to give more meaning and understanding to the research topic.^21^ Lastly, the contextual design focused on the participants’ natural settings.^22^ This design allowed the researcher to capture the essence of family members’ experiences of having a relative admitted as a state patient in the comfort of their homes. To that end, the researcher employed a phenomenological research method.^23^

### Setting

Family members were identified through their relatives who were admitted to a psychiatric hospital in Gauteng. However, the study was conducted in the participants’ homes. There was no specific area that was aimed at. Participants were from different townships in Gauteng, Soweto and Mamelodi. The family members were characterised by unemployment and poverty. They came from formal and informal settlements. There was access to schools and healthcare centres; however, sewage drainage and garbage collections were poorly serviced.

### Research population and sampling strategy

The research population was family members of an individual admitted as a state patient. Participants were selected from this population using purposive sampling.^24^ The inclusion criteria were relatives of individuals admitted to a psychiatric hospital for less than 2 years as state patients. Participants were any family member who formed ties with the state patient and was affected by their admission. It was either parents, spouses, siblings or children older than 18 years. Participants also had to be able to speak English, Setswana or isiZulu and willing to sign informed consent forms. The sample size was determined during data collection when saturation^24,25^ was reached at the seventh interview^21,26,27^; at this point, data repeated itself, and no new information was found.^28^

### Data collection

Raw data were collected using more than one technique.^20^ In-depth phenomenological interviews, field notes and observations were intentionally employed to achieve the purpose of the study. All interviews were opened with the central question: ‘How is it for you to have a relative admitted as a state patient?’ This was followed by subsequent probing questions guided by participants’ responses to the initial question. Exploring, paraphrasing and clarification were also employed to prompt participants’ expression of feelings and thoughts about their experiences. Six interviews were conducted in Setswana (the researcher is proficient in Setswana), and one was conducted in English. A digital voice recorder was used, and consent was requested to audio-record the conversation before data collection commenced. Interviews were approximately 40 min–60 min each. The data collection process took over 4 months, from February 2021 to May 2021. At that time, the country was under lockdown level two because of the coronavirus disease 2019 (COVID-19) pandemic; this allowed face-to-face interviews, which ensured the study’s credibility and the use of all senses for observation during data collection.^22^

### Data analysis

Data collection and analysis co-occurred. The recordings were transcribed, and the researcher translated Setswana interviews into English. Data analysis entailed the organisation, reduction and transformation of the data by exploring the meaning of what was gathered.^29,30^ Moreover, Tesch’s thematic coding method^20^ was used to analyse data. Recorded interviews were thus listened to, and transcriptions and field notes were read to get a sense of the whole. One interview was focused on at a time, and this analysis method helped the researcher to explore participants’ meanings and group raw data into smaller units to identify essential themes. These units were categorised into three themes. The researcher and an independent coder (an expert in qualitative research) analysed the transcribed data separately.

### Rigour

The researcher maintained a researcher’s role while recruiting participants, as she was employed in the same hospital as admitted relatives. The researcher implemented Guba’s model of trustworthiness^31^ and focused on the findings’ credibility, confirmability, dependability and transferability to promote the study’s quality. The researcher used data triangulation to promote the credibility of the study by using in-depth phenomenological interviews, field notes and observations as data collection methods. Confirmability was met by virtual meeting with an independent coder to discuss the emerging themes, and consensus was reached on the main themes and meaning units to ensure the trustworthiness of the analysed data. Dependability was met by an independent coder analysing data separately from the researcher. A deeper understanding of the phenomenon was provided using direct quotes from the participants to ensure transferability and confirmability.

### Ethical considerations

All procedures performed in studies involving human participants should be in accordance with the ethical standards of the institutional and/or national research committee, the 1964 Helsinki Declaration and its later amendments or comparable ethical standards. This study received approval from the Research Ethics Committee of the Faculty of Health Sciences (reference no.: REC-650-2020), the psychiatric hospital and the Higher Degrees Committee (reference no.: HDC-10-58-2020). Written informed consent was also obtained from all individual participants involved in the study, whose participation was voluntary. The researcher kept participants anonymous, no harm was inflicted, all participants were fairly treated and the study aimed to promote the interests of the participants. The recorded interviews, transcripts and reports were stored in a computer file and secured with a password. This data will be kept for 2 years after the study’s publication in an accredited journal.

## Results

Following data collection, the following were gathered. Seven interviews were conducted; out of seven, three sessions of interviews, the participants were in pairs and the other four sessions were sitting alone for interviews. There were 10 participants overall, five males (father, two uncles, husband and a brother) and five females (mother, two sisters, aunt and a niece). The age group ranged between 31 years and 59 years. The relatives have been admitted for less than 10 months. The participants’ characteristics are represented in Table 1.

**TABLE 1:** Participants’ characteristics.

Interview	Participant number	Gender	Relationship to the relative	Age	Months relative admitted as a state patient
1	P1	Female	Mother	50 years	3 months
	P2	Male	Father	59 years	3 months
2	P3	Male	Uncle	59 years	9 months
3	P4	Male	Husband	37 years	4 months
4	P5	Female	Sister	48 years	2 months
5	P6	Male	Uncle	50 years	3 months
	P7	Female	Aunt	42 years	3 months
6	P8	Male	Brother	40 years	3 months
7	P9	Female	Sister	54 years	2 months
	P10	Female	Niece	31 years	2 months

*Source*: Malebye SS. Experiences of family members of relatives who have been admitted as state patients in a psychiatric hospital in Gauteng Province [homepage on the Internet]. University of Johannesburg; 2021. Available from https://ujcontent.uj.ac.za/esploro/outputs/graduate/Experiences-of-family-members-of-relatives/9910917007691#file-0^32^

Note: Participants’ numbers are used to protect their identity.

Three themes emerged from the analysed data: (1) family members initially experienced negative feelings towards their relative that later turned into compassion; (2) family members experienced a sincere desire to support their relative but struggled with some barriers within the system and (3) family members experienced a great need to impart information and knowledge about mental illness to the community to protect and assist their vulnerable relative.

### Family members initially experienced negative feelings towards their relative that later turned into compassion

Participants experienced common feelings of anger and fear when their relative’s behaviour changed, and they started showing signs of mental illness. These relatives’ behaviour continuously worsened, and participants shared that they were confused about what was happening to their relatives. Behavioural changes were painful and brought about anger as participants did not understand what was happening. Some participants still expressed anger on the day data were collected. One participant expressed:

‘Ne re kwatile pele re bona the way a treatang batho ka teng, ebe a etsa ntho a editseng, maar hantse re ya re bona hore ho tjhong motho o nasa iketse. [*We were angry at first seeing the way he treated people, then he did what he did, but when time went, we saw that this person wasn’t doing this on purpose*.]’ (Interview 7, P9, 54 years, sister)

The anger participants experienced was also exacerbated by not knowing the cause of the sudden change in behaviour and thinking that their relative’s behaviour was deliberate. One participant described the fear she experienced as a result of her relative’s behaviour:

‘O na lwana ka tlung ka mona, ele motho ere motshabang, o na tsosa, hehhh [expression] motho eo na tshosa, re mo tshaba. [*He was fighting in the house, he was the person whom we were afraid of, he was scary, hehhh that person was scary, we were afraid of him.*]’ (Interview 1, P1, 50 years, mother)

Participants said they did not know the initial signs of mental illness, which gave rise to their anger and fear, anger from not knowing what was wrong with the relative and thinking they were acting out of character purposefully. Some participants also feared for their safety and had to be guarded in what they said to their relatives because of their unknown reactions, as indicated by Participant 1.

Participants developed compassion by seeking help from social worker, the South African Police Service and community healthcare centres as soon as they realised their relatives were mentally ill. Anger and fear transitioned into positive feelings of relief and acceptance. A feeling of relief was experienced as soon as participants realised their relatives would receive help once they were admitted to the hospital. This automatically created feelings of acceptance among participants that their relative had a mental illness.

One participant explained why she felt relieved about her relative’s admission by saying:

‘I’m feeling positive about him being in hospital because he will really get some help’ (Interview 7, P10, 31 years, niece)

Participant 8 also explained that his anger transitioned to acceptance:

‘ke ile ka mokwatela ebe ka itjwetsa hore motho enwa ke ngwana heso, o nale bothata, o hloka thuso e right. [*I was angry at him and told myself that he is my sibling, he has a problem, he needs a serious help*.]’ (Interview 6, P8, 40 years, brother)

Participants seemed to understand that their relative’s admission to a psychiatric hospital would create a change in their behaviour. They accepted that their relatives were mentally ill and sought help to get them admitted. Their relative’s admission to a psychiatric hospital brought relief and a sense of serenity.

### Family members experienced a sincere desire to support the relative but struggled with some barriers from police services and healthcare facilities

Despite how they initially felt about them, all participants expressed their caring through the kind of support they offered and wanted to give their relatives so that they could return home again. However, they experienced barriers from the police services and healthcare facilities that made it challenging to support their relatives, such as getting them admitted promptly, no communication from healthcare workers and visiting restrictions during the COVID-19 pandemic.

Participant 5 acknowledged that her relative had full support from home and that they showed it through telephone calls.

One participant explained how she showed support to her relative by stating:

‘He has a supportive structure, and he is happy that we are keeping touch with him.’ (Interview 4, P5, 48 years, sister)

Two participants shared the same feeling of frustration towards the slow services that their relatives delayed receiving. Participant 5 shared that, even though she remained supportive, the system may have played a role in contributing to her relative’s mental illness. She expressed her frustration as follows:

‘Hmmmm five years on trial? It’s not fair, why couldn’t they have picked it up hore [*that*] he is not fit for trial? Only after five years. A lot can happen in five years, frustration, breakdown.’ (Interview 4, P5, 48 years, sister)

To add to the slow admission process, COVID-19 caused a barrier for three participants to visit their relatives. All participants had the means to communicate with their relatives as they could not see them in the hospital and to continue showing support.

Participant 1 described how the pandemic prevented them from visiting the relatives:

‘Ne re batla le ho motjhakela, abare re ka se kgone ho tla ka baka la COVID, lona le ka nromella tjhelete feela. [*We wanted to visit him, he said we won’t be able to due to COVID, we could just send him money*.]’ (Interview 1, P1, 50 years, mother)

Participant 5 acknowledged that her relative had full support from home and that they showed it through telephone calls:

‘Ra bua founung but ga gotshwane le fa leteng mo gae. [*We talk on the phone but it’s not the same when he is at home.*]’ (Interview 4, P5, 48 years, sister)

Participants expressed a need for feedback from healthcare providers. There was also a need for participants to be included in decisions about their relative’s care and treatment. One family member explained why he thought feedback was important:

‘Yona e bohlokwa haholo, akere re mo isitse moo ka concern, re lebelletse ho tseba, jwalo ka ha re lebelletse di outcome tsa bona re lebelletse ho tseba hore nah according to bona process etlo nkuwa ke efeng and then etlo mo thusa how far. [*It is very important, we took him there with concern, we are looking forward to knowing, as much as we are looking forward to their outcomes, we are looking forward to know whether according to them what process will be taken and how far will it help him.*]’ (Interview 5, P6, 50 years, uncle)

Participant 5 explained how families should be included in decisions:

‘I think families should be considered *hore bona ba batla* [*on what they want*], they should be informed and included *ko di* [*in*] decisions that they take as *court le spetlele* [*court and hospital*].’ (Interview 4, P5, 48 years, sister)

As participant 5 mentioned, family members needed to know what was happening with their relatives and their prognosis after hospital admission. It sounded as if they wanted to know each step taken to care for their relative. In addition, participant 4 wanted to be included in the decisions taken for and on behalf of their relative from the court proceedings until admission to the hospital.

### Participants experienced a great need to impart information and knowledge about mental illness to the community to protect and assist their vulnerable relatives

Participants showed a great desire for healthcare providers to share information not only with them but with the community as well. They believed that imparting knowledge to the public was vital and somehow would protect their relatives when they returned to the community.

Participant 3 explained what he knew versus what he learned in referring to a person with mental illness:

‘Ne re tseba hothwe motho wa hlanya eseng mental illness. [*We knew that a person was crazy not mental illness*.]’ (Interview 2, P3, 59 years, uncle)

A mother also explained why she felt her relative was safer in the hospital rather than being out in the community:

‘Ke kgotsofaditswe ke hore o dutse moo ho leng safe ho fapana le ha nale hae. [*What is making me more satisfied is that where he is, it is safe as compared to when he was home.*]’ (Interview 1, P1, 50 years, mother)

It seemed that all participants experienced mental illness for the first time with their relatives when they became ill. Participant 3 indicated that he did not know a polite way of referring to people with mental illness. Furthermore, the lack of knowledge about mental illness meant participants felt their relatives were safer in the hospital than out in the community.

Even though participants needed information about their relative’s progress, they also had other means to cope in their daily lives. Four participants used hope and prayer for the recovery of their relatives.

The following participant was quoted saying:

‘Empa ka baka la hore ra tseba hore moo a ileng olo thola thuso, re be le tshepo hape hore ho tlo loka hobane ntho engwe le engwe enale nako, re dutse feela tshepong. [*Because we knew where he went, he will get help, we have hope and that he will be good because everything has its own time, we are sitting on hope.*]’ (Interview 5, P7, 42 years, aunt)

Participant 4 and participant 8 shared how spiritual support may help them cope:

‘*Ke nahana re hloka* [*I think we need*] spiritual support because there are situations that a human being cannot control like *yona ena* [*this one*], I think God will guide us.’ (Interview 3, P4, 37 years, husband)

‘Ho tseba ntate modimo hore ho tla etsahala eng, se ke beile Tshepo ya ka ho yena. [*Only God knows what will happen, I have put my trust on him.*]’ (Interview 6, P8, 40 years, brother)

Participant 7 indicated that she knew where her relative was, which gave her peace. She said she would hold on to hope for recovery when the time comes. Participant 4 and participant 8 both indicated that they needed spiritual support and for God to take control of the situation beyond their control. They, therefore, did not only rely on the MDT to help their relatives but also their faith.

## Discussion

The themes were derived from the theoretical framework of Kubler-Ross. This theoretical framework acknowledges that when one is experiencing loss in their life, they will process the loss through various stages. Family members of relatives admitted as state patients in a psychiatric hospital indicated that their initial response to the unknown was negative. However, as soon as they learned the cause of their relative’s change in behaviour, compassion developed. As much as they wanted their relatives to be admitted to the hospital, they also wanted them to return home after healing and rehabilitation.

Literature indicated that anger is a normal response to grief.^33^ As family members initially felt their relatives were pretending to be mentally ill, they responded with anger. Anger is a common response among families before mental illness is diagnosed.^34,9^ The anger experienced was accompanied by fear, which has been classified as a negative emotional expression.^35^ Participants feared their relatives because they displayed violent behaviour; they feared being attacked and exposed to danger and injuries.^36^

Positive feelings bloomed when compassion was experienced and participants started to accept that their relatives had a mental illness. They sought help from social workers, the SAPS, and the community healthcare centre. In the last stage of grief (acceptance), families finally understand that their relatives have a mental illness.^33,9^ Participants were relieved when their relatives were admitted to a psychiatric hospital to receive help. This finding is also supported by a study that reported the hospital admission of a mentally ill relative brought relief from unnecessary expenses that were incurred when individuals destroyed property during the early days of mental illness.^3^ The transition of negative feelings into positive affective responses promoted participants’ desire to support their relatives. The manner in that all participants experienced their feelings supports the model of stages of grief, even though not all stages were experienced in the same pattern.

The findings indicated that participants still cared for their relatives and continued to support them. Physical and non-contact support increases the relative’s treatment response in the hospital.^37,38^ Physical contact was restricted because of COVID-19 regulations,^39^ but families maintained telephonic contact with their relatives. Literature similarly found that communication is vital to the admitted relative, especially when geographical distance forms a barrier for families to visit.^40^ The COVID-19 pandemic ultimately limited visitation.

During data collection, it was evident that participants did not understand the process that led to an individual becoming a state patient and did not understand the meaning of the term itself. This showed a gap in communication between healthcare providers and the families, creating a barrier and preventing families from effectively supporting their relatives.^41^ A lack of support from the MDT caused families to feel lonely and feel abandoned by the team. Furthermore, the participants recommended a comprehensive programme for healthcare providers to meet the needs of families, where information, feedback and conversations regarding their experiences could be explored. Information should also be shared with the community because families were worried about their relatives’ safety upon their return. In addition, participants not only wanted to be informed but also wanted to be part of the decision-making process.^42^

The study revealed that participants had a solid Christian spiritual belief system. They remained connected to supporting church organisations and ministerial services. Prayer and faith were observed to promote self-actualisation and enhanced the families’ ability to support their relatives admitted as state patients.^7,9^

### Limitations

The study’s limitations are that the researcher worked in the same psychiatric hospital as admitted relatives, which may have influenced access to the family members’ contact details and their willingness to participate. The role of the researcher was misunderstood as that of being an employee of the institution where their relatives were admitted to. The researcher had to explain and emphasise that she was in the capacity of a researcher. There are limited studies about families with relatives who are state patients in South Africa, and no statistics as well. The background of the study was therefore supported by limited research from the country.

### Recommendations

The researcher strongly suggests a prompt therapy session with families when mental illness is diagnosed to clear the frustrations and shock. Mental health clinics should be integrated with the primary healthcare centre to increase community awareness and accessibility and potentially decrease stigmatisation. Media should be used to access a larger population to promote mental health awareness. A support system should be fostered that encourages and respects family decisions in the interest of loved ones’ treatment plan.

## Conclusion

The study aimed to explore and describe the experiences of family members with a relative admitted as a state patient in Gauteng province. Data revealed that participants experienced similar fear at the beginning of their relatives’ mental illness, which transitioned to the need for their relatives to receive help. Barriers within the healthcare system and police services did not obstruct them from getting their relatives appropriate help. Participants were of the opinion that their involvement in their relative’s treatment plan could promote their relative’s response to treatment. Furthermore, their relative’s safety might be guaranteed when they return home, provided the community is informed about mental illness.

The researcher discovered that families support their relatives; they are willing to show them affection.^33^ Their contribution to the decision-making process would improve long hospital stays. None of the participants resented their relatives despite the reason for being admitted as state patients. Furthermore, information on mental health and illness can easily reach the community through family members as long their mental health is cared for.
